# Prices of second-line antiretroviral treatment for middle-income countries inside versus outside sub-Saharan Africa

**DOI:** 10.7448/IAS.17.4.19604

**Published:** 2014-11-02

**Authors:** Bryony Simmons, Andrew Hill, Nathan Ford, Kiat Ruxrungtham, Jintanat Ananworanich

**Affiliations:** 1 School of Public Health, Imperial College London, London, UK; 2 Molecular and Clinical Pharmacology, University of Liverpool, Liverpool, UK; 3 Department of HIV/AIDS, World Health Organisation, Geneva, Switzerland; 4 Department of Medicine, Chulalongkorn University, Bangkok, Thailand; 5 Military HIV Research Program, Walter Reed Army Institute of Research, Silver Spring, MD, USA

## Abstract

**Introduction:**

Antiretrovirals are available at low prices in sub-Saharan Africa, but these prices may not be consistently available for middle-income countries in other regions with large HIV epidemics. Over 30% of HIV infected people live in countries outside sub-Saharan Africa. Several key antiretrovirals are still on patent, with generic production restricted. We assessed price variations for key antiretroviral drugs inside versus outside sub-Saharan Africa.

**Methods:**

HIV drug prices used in national programmes (2010–2014) were extracted from the WHO Global Price Reporting Mechanism database for all reporting middle-income countries as classified by the World Bank. Treatment costs (branded and generic) were compared for countries inside sub-Saharan Africa versus those outside. Five key second-line antiretrovirals were analysed: abacavir, atazanavir, darunavir, lopinavir/ritonavir, raltegravir.

**Results:**

Prices of branded antiretrovirals were significantly higher outside sub-Saharan Africa (p<0.001, adjusted for year of purchase) (see Table 1). For example, the median (interquartile range) price of darunavir from Janssen was $732 (IQR $732-806) per person-year in sub-Saharan Africa versus $4689 (IQR $4075-5717) in non-African middle-income countries, an increase of 541%. However, when supplied by generic companies, most antiretrovirals were similarly priced between countries in sub-Saharan Africa and other regions.

**Conclusions:**

Pharmaceutical companies are selling antiretrovirals to non-African middle-income countries at prices 74–541% higher than African countries with similar gross national incomes. However, generic companies are selling most of these drugs at similar prices across regions. Mechanisms to ensure fair pricing for patented antiretrovirals across both African and non-African middle-income countries need to be improved, to ensure sustainable treatment access.

**Table 1:** Comparison of the cost of antiretroviral treatment, by manufacturer and region

Manufacturer	ARV (dose)	No. of Sub-Saharan Africa countries (no. of individual transactions)	Sub-Saharan Africa median cost per person per year (IQR), US$	No. of Non-Africa countries (no. of individual transactions)	Non-Africa median cost per person per year (IQR), US$	Price rise, non-Africa vs. Sub-Saharan Africa (%)
Branded	ABC (300 mg)	2 (93)	315 (294–315)	3 (10)	547 (299–602)	74
	ATV (300 mg)	2 (170)	357 (124–357)	2 (4)	1910 (1910–3496)	435
	DRV (600 mg)	7 (84)	732 (732–806)	9 (31)	4690 (4075–5717)	541
	LPV/r (200 mg/50 mg)	15 (492)	319 (272–374)	23 (128)	720 (456–932)	125
	RAL (400 mg)	3 (52)	883 (883–1010)	1 (2)	3589 (3589–3589)	306
Generic	ABC (300 mg)	18 (290)	192 (167–213)	33 (215)	178 (155–205)	−7
	ATV (300 mg)	6 (34)	296 (251–309)	14 (36)	245 (219–265)	−17
	DRV (600 mg)	2 (2)	990 (964–1016)	1 (1)	2964 (2964–2964)	199
	LPV/r (200 mg/50 mg)	18 (164)	391 (282–429)	33 (187)	397 (349–435)	2
	RAL (400 mg)	2 (28)	373 (373–634)	0	–	–

